# Cervical leukoplakia in Ghana: Unveiling diagnostic challenges and informing low-resource screening strategies

**DOI:** 10.1371/journal.pone.0345847

**Published:** 2026-09-09

**Authors:** Kofi Effah, Ethel Tekpor, Maxwell Afetor, Joseph Emmanuel Amuah, Comfort Mawusi Wormenor, Annita Edinam Dugbazah, Stephen Danyo, Seyram Kemawor, Edna Sesenu, Christopher Nyamekye, Emmanuel Timmy Donkoh, Chrysantus Kubio, Kofi Adum-Attah

**Affiliations:** 1 Cervical Cancer Prevention and Training Centre, Catholic Hospital, Battor, Ghana; 2 Department of Obstetrics and Gynaecology, Catholic Hospital, Battor, Ghana; 3 Department of Epidemiology and Biostatistics, Fred N. Binka School of Public Health, University of Health and Allied Sciences, Ho, Volta Region, Ghana; 4 Volta Regional Health Directorate, Ghana Health Service, Ho, Volta Region, Ghana; 5 School of Epidemiology and Public Health, Faculty of Medicine, University of Ottawa, Ottawa, ON, Canada; 6 University of Ghana Medical Centre, Accra, Ghana; 7 Central Laboratory, Catholic Hospital, Battor, Ghana; 8 Screen-and-Treat Research Group, Centre for Research in Applied Biology, School of Sciences, University of Energy and Natural Resources, Sunyani, Ghana; 9 Department of Family Medicine, Cape Coast Teaching Hospital, Cape Coast, Ghana; Teikyo University: Teikyo Daigaku, JAPAN

## Abstract

**Background:**

Visual Inspection with Acetic acid (VIA) is widely used for detecting cervical (pre)cancer in resource-limited settings due to its simplicity and cost-effectiveness. However, the finding of cervical leukoplakia, poses diagnostic challenges.

**Methods:**

This retrospective observational study evaluated the prevalence, associated risk factors, and referral outcomes of cervical leukoplakia among 13,861 women previously screened for cervical cancer in Ghana between June 2016 and May 2024. Screening approaches included VIA alone, mobile colposcopy alone, or VIA/mobile colposcopy combined with other tests (HPV DNA testing and/or cytology).

**Results:**

Among 120 women (0.9%) with leukoplakia, 112 (93.3%) did not have acetowhitening on VIA/mobile colposcopy, and 21 (17.5%) received LEEP. The histopathology results ranged from no Cervical Intraepithelial Neoplasia (CIN) (15) to CIN I (2), CIN II (1), and CIN III (3). High-risk HPV positivity (aOR: 30.27; 95% CI: 3.56–257.37) and HIV infection (aOR: 8.27; 95% CI: 1.18–57.69) were associated with higher odds of leukoplakia in VIA-screened women.

**Conclusion:**

Leukoplakia can be clinically important without coexistence with acetowhitening. The association of leukoplakia with hrHPV and HIV, coupled with a high referral default and logistical barriers to biopsy and histopathology, calls for simplified management protocols and point-of-care triage tools for cervical screening in low-resource settings.

## Introduction

Cervical cancer remains a major contributor to cancer-related mortality in women worldwide, particularly in low- and middle-income countries (LMICs) [1]. In Ghana, it is the second most prevalent malignancy among women, with an annual incidence of 2,797 cases and 1,699 associated fatalities [1]. The disproportionate mortality stems from systemic challenges that delay recognition of precursor lesions and limit access to prompt treatment, both essential for attaining disease elimination targets [2–5]. Timely identification of cervical precancerous lesions holds promise for reducing the risk of cancer. In low-resource settings, cytology-based screening faces significant obstacles, including shortages of trained personnel, inadequate infrastructure, and high costs [6]. As a result, visual inspection methods are a pragmatic choice for expanding access to screening services because of their affordability and accessibility.

Leukoplakia, a non-scrapable white lesion visible on the cervix before acetic acid application, is a common finding during Visual Inspection with Acetic acid (VIA) or colposcopy [7,8]. Unlike acetowhite lesions, leukoplakia is discernible without acetic acid and may indicate underlying high-grade lesions or invasive pathology [9]. It may result from hyperkeratosis, chronic inflammation, or HPV-related epithelial changes. However, its opaque appearance can obscure underlying high-grade Cervical Intraepithelial Neoplasia (CIN2/3) or invasive carcinoma and is associated with a higher likelihood of concealing severe dysplasia or malignancy on biopsy [10,11]. This diagnostic challenge underscores the importance of histological evaluation in cases of leukoplakia to rule out serious pathology, particularly in settings where advanced imaging or molecular testing is limited [11–13]. The clinical significance of leukoplakia has been well-documented in other mucosal tissues; however, its diagnostic importance in cervical screening is insufficiently explored [9]. Research indicates varying relationships between leukoplakia and cervical (pre)cancer or cancer, shaped by population-specific factors, including HPV genotype distribution, immune status, and healthcare access [10,13–15]. These inconsistencies highlight the need for further evidence to clarify the interplay between leukoplakia and established predictors of cervical intraepithelial lesions.

Despite the widespread use of VIA, the clinical significance and management of leukoplakia, particularly in diverse resource-limited settings like Ghana, remain under-explored. Consequently, there is a paucity of literature on the significance of leukoplakia in cervical precancer screening to guide local screening programmes as current guidelines do not explicitly advance specific recommendations for leukoplakia management in basic and limited healthcare settings. Therefore, we conducted a retrospective analysis of existing data to investigate the impact of visual screening approaches (VIA/mobile colposcopy) on the reporting of leukoplakia and real-world referral outcomes among women previously screened for cervical cancer in primary care contexts in Ghana. We also investigated the cross-sectional factors associated with leukoplakia among women and reflect on the clinical and public health significance of leukoplakia in the present context to inform and improve cervical cancer screening approaches in resource-constrained settings.

## Methods

### Study design and setting

Screening data were collected as part of routine service delivery from June 2016 to May 2024 at the Catholic Hospital, Battor. In order to understand the distribution of leukoplakia findings according to screening (VIA/mobile colposcopy) approaches typically used in low-resource settings, we performed a retrospective examination of screening records; data retrieval and statistical analysis was conducted from March to October 2025. As previously indicated [16], the CCPTC is a primary screening facility for cervical cancer located in the Eastern Corridor of Ghana, specifically in the Volta Region. The Region is home to a total population of 1,659,040 inhabitants and is located westward of the Republic of Togo and extending to the Volta River. The CCPTC is situated on the premises of the Catholic Hospital, Battor, Ghana in the North Tongu District of the Volta Region and is synonymous with skills development for cervical cancer prevention in Ghana. The Centre was founded in 2017 with the core mandate of training health workers to provide cervical precancer screening services in their host institutions, as well as generating empirical data to inform cervical cancer policy development. The CCPTC operates through a hub-and-spokes model [17] employing centralized and mobile services to extend cervical cancer preventive services to adjacent communities [18]. Over the years, approximately 60% of women attending the CCPTC for screening services reside in Greater Accra Region, 30% in the Volta Region (in which the Centre is located), and 10% in the Eastern Region. This report covers both women evaluated via outreach initiatives and those who attended the CCPTC for screening services using a uniform guideline-based approach [3,19].

### Study participants and sampling

A convenient sampling of all complete records of women aged 18 and above who attended routine cervical cancer screening via hospital visits and mobile outreach clinics and were eligible for inclusion in the present study was conducted. Cervical precancer screening with HPV DNA testing is performed for women 25 years and above in routine screening at the CCPTC. For cytology and visual inspection methods (VIA and mobile colposcopy), screening begins at 21 years old. However, on some occasions, especially for parous women less than 21 years old, screening was offered as long as women were counselled and understood the benefits and harms of screening. Identified records were mostly women from communities in the Volta Region who were interviewed and screened by or under the supervision of experienced CCPTC trainees (nurses). Records of participants who received cervical screening via VIA only, mobile colposcopy only, or a combination of VIA with additional tests (HPV DNA testing and/or cytology) were included. The main exclusion criterion was incompleteness of medical records.

### Data collection

Data retrieved from the medical records of all women screened from June 2016 to May 2024 included patient de-identified demographics (age, parity, HIV status), screening results (presence of leukoplakia, acetowhite lesions, and other cervical abnormalities), and follow-up data. Details regarding cervical screening procedures, such as cervical specimen collection, Visual Inspection with Acetic acid (VIA and EVA (Enhanced Visual Assessment using mobile colposcopy)), and hr-HPV DNA testing used during primary data generation have been previously published [20–26]. Additionally, the research team retrieved anonymised data on women referred for further specialist investigation and management, including women who failed to complete follow-up, along with those unable to afford histopathology services [27].

### Cervical screening procedures: Cervical specimen collection, Visual Inspection with Acetic acid (VIA and EVA mobile colposcopy), and hr-HPV DNA testing

**Fig 1:**
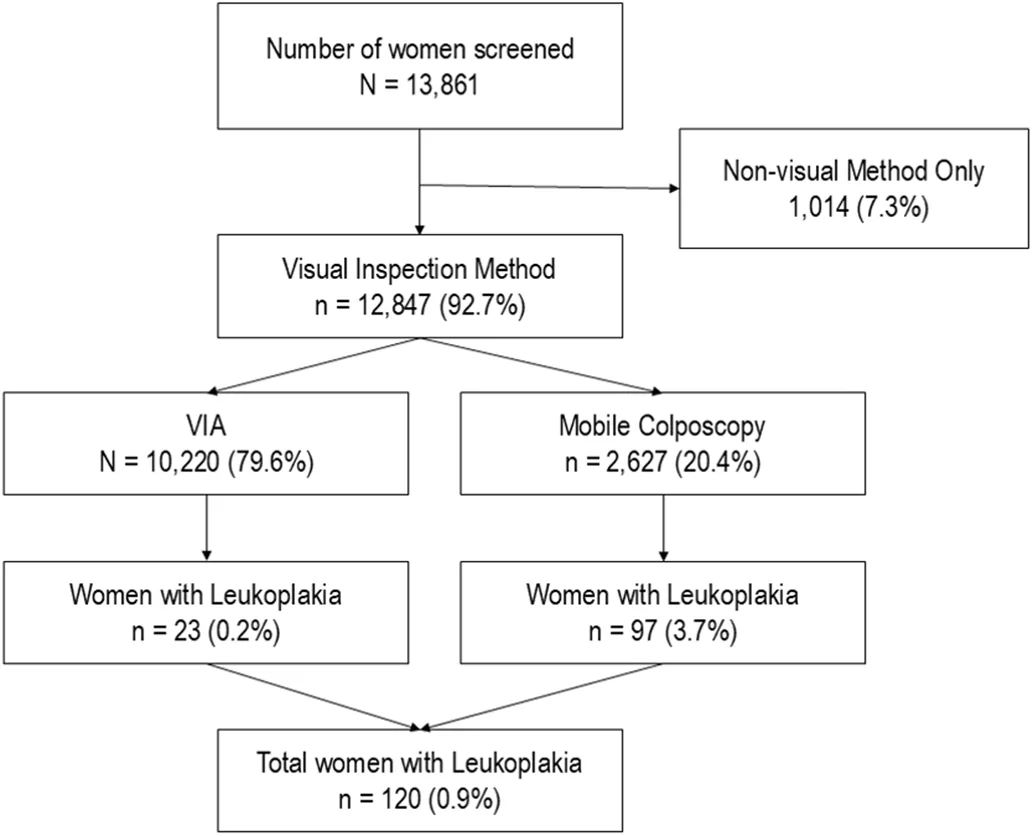
Study flowchart showing screening techniques and primary screening outcome. A total of 13,861 women were screened. Of these, 12,847 underwent visual inspection (VIA or enhanced visual assessment [EVA]/mobile colposcopy) with leukoplakia assessment, while 1,014 were screened using non-visual methods (HPV DNA testing and/or cytology) without leukoplakia assessment. Overall leukoplakia prevalence was 0.9% (n = 120/13,861; 95% CI: 0.7–1.1). Prevalence was 0.2% (n = 23) among VIA-screened women and 3.7% (n = 97) among EVA-screened women.

Per the established protocol for screening at the CCPTC, each woman received counselling regarding the benefits and potential risks associated with cervical precancer screening. The combination of screening test(s) offered was determined by several contextual factors, including the screening setting, and involved three main approaches: standalone testing (using HPV DNA testing, *or* VIA, *or* EVA mobile colposcopy), *or* concurrent testing (HPV DNA testing + any visual inspection procedure in a single session), *or* tritesting (HPV DNA testing + any visual inspection method + Pap smear in a single session). Women who had only non-visual testing results were not included in the present report (Fig 1). Visual assessment-based screening was carried out with the woman lying in the dorsal lithotomy position and standard speculum placement to achieve cervical exposure. Cervical specimens for hr-HPV DNA testing were routinely taken by sampling the transformation zone (TZ) with a cytobrush or cotton-tipped applicator. The specimens were then placed in a collection tube, sealed, stored dry, and sent to the laboratory for processing and testing within one week.

Among women who underwent Pap smear screening, cervical samples were collected using either a Cervex-Brush® for liquid-based cytology (LBC) or an Ayre spatula and cytobrush for conventional cytology. The preservativemeduim for LBC was PreservCyt (ThinPrep), while the conventional samples were smeared on a glass slide and fixed with 92% ethyl alcohol. During sampling for conventional cytology, additional samples were taken for HPV DNA testing using a cytobrush or cotton-tipped applicator. For liquid-based cytology, a portion of the sample (16 mL) was sent for testing. The choice of cytology method depended on kit availability, with a preference for liquid-based cytology. Cytology results were available within two weeks after evaluation by a pathologist offsite.

For women who were screened by visual inspection methods, VIA and/or EVA mobile colposcopy was performed after collecting cytology and HPV DNA samples. VIA was performed, as per standard procedure by examining the cervix under a good light source, applying 5% acetic acid and waiting for 1.5 to 2 minutes. Generally, the EVA system 3.0 (MobileODT, Tel Aviv), a mobile colposcope that attaches to a smartphone, was used to capture images before and after applying acetic acid for quality assurance purposes, enabling retrospective adjudication. Colposcopy findings were reported in line with the International Federation of Cervical Pathology and Colposcopy (IFCPC) terminology [28], noting the adequacy of the procedure, the type of TZ, and any significant cervical lesions. Visual inspection results were classified as positive or negative based on the presence of abnormal acetowhite lesions.

### Laboratory processing of cervical specimens for hr-HPV DNA testing

The choice of assay for HPV DNA analysis of cervical specimens depended on the testing platform in use at the laboratory of the Catholic Hospital, Battor, at the time of specimen submission. Four test systems were available: careHPV (Qiagen GmBH, Hilden, Germany), GeneXpert HPV test (Cepheid, Sunnyvale, CA, USA), the AmpFire HPV DNA test platform (Atila BioSystems, Inc., Mountain View, USA), or MA-6000 HPV assay system (Sansure Biotech Inc., Hunan, China). All tests were run at the central laboratory or CCPTC laboratory of Catholic Hospital, Battor, in strict adherence with each manufacturer’s guidelines and protocols, as detailed in previous studies [20–26]. Given resource constraints and the need to sustain screening activities, multiple platforms were used across calendar time and settings. For careHPV, we used a dry brush (the careBrush) transported in a tube with a proprietory buffer. For GeneXpert, we used a brush, the Cervex brush, transported in PreservCyt/ThinPrep. AmpFire and MA-6000 assays worked with cervico-vaginal swabs (dry brushes, swabs, or PreservCyt/ThinPrep). CareHPV identifies 14 high risk HPV – HPV 16, 18, 31, 33, 35, 39, 45, 51,52, 56, 58, 59, 66, and 68 (as positive or negative, not individually). The GeneXpert also identifies 14 high risk HPV – HPV 16, 18/45 (specific identification) and 11 other hr-HPV type(s): 31, 33,35, 39, 51, 52, 56, 58, 59, 66, 68. The semi-quantitative modules of the AmpFire and MA-6000 separately and specifically identify HPV16 and HPV18, while collectively identifying HPV31/33/35/39/45/51/ 52/53/56/58/59/66/68 as *other* high-risk HPV types.

### Case management strategy and follow-up

**Fig 2:**
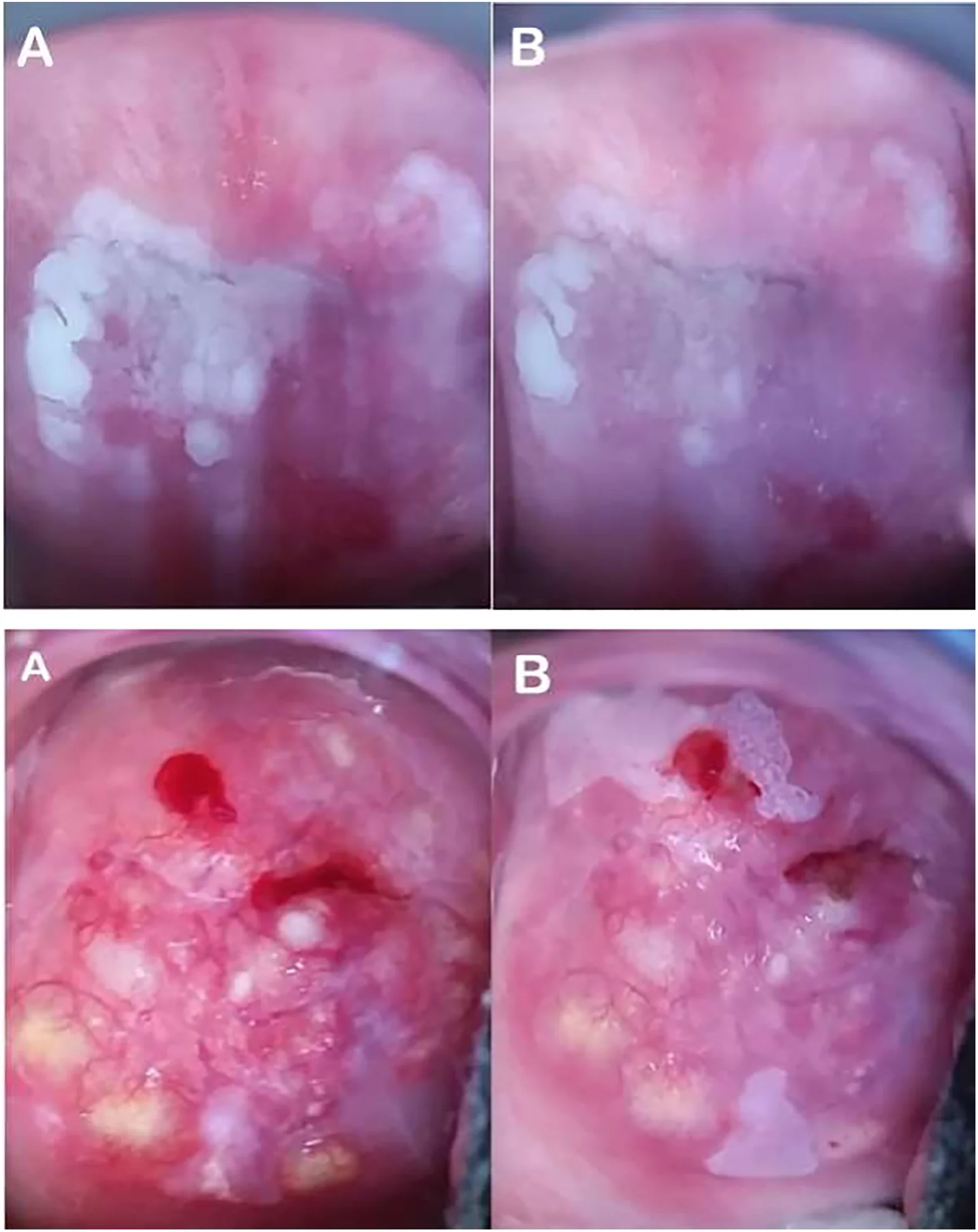
Representative colposcopic images obtained before (A) and after (B) acetic acid application. Top row (Patient 1) and bottom row (Patient 2) show thick, non-scrapable white plaques visible prior to acetic acid application with little to no acetowhitening afterward. These images illustrate the characteristic appearance of leukoplakia, which can obscure underlying high-grade Cervical Intraepithelial Neoplasia or invasive carcinoma and poses significant diagnostic challenges during visual inspection screening in low-resource settings.

**Fig 3:**
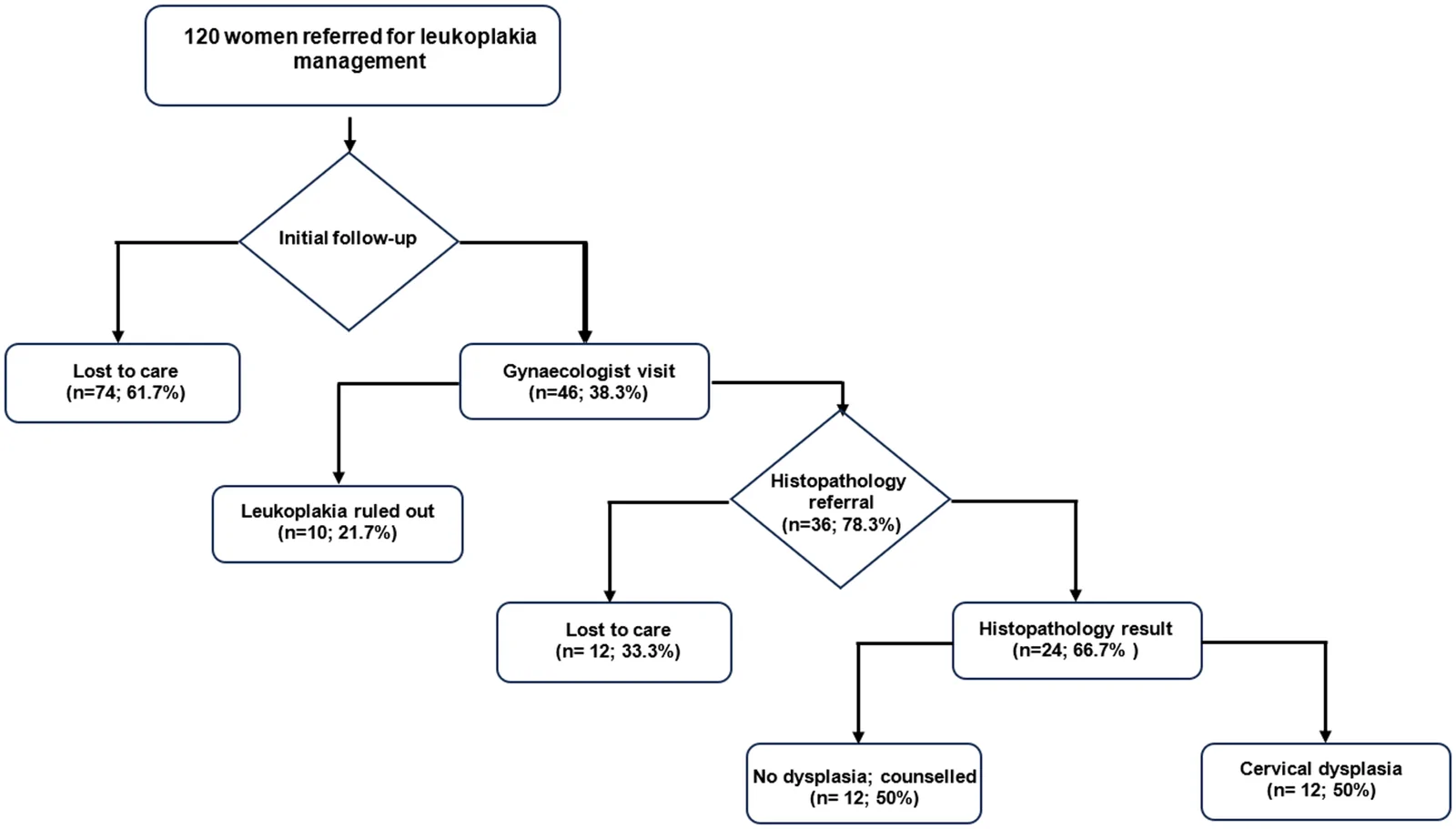
Referral cascade and outcomes for 120 women diagnosed with cervical leukoplakia. Seventy-four women (61.7%) were initially lost to care. Of the 46 who attended gynecologist review, leukoplakia was ruled out in 10 (21.7%) and biopsy/LEEP was recommended for 36. Of these 36, 12 were subsequently lost to care; 24 completed histopathology (no dysplasia: 12; CIN I: 3; CIN II: 5; CIN III: 4). No invasive cancer was found. Overall, 96/120 (80%) did not complete biopsy/LEEP.

Given the resource constraints and the risk of losing clients to follow-up, a screen-and-treat approach was employed as much as possible in line with recommendations for screening in resource-limited settings [29]. Details of the case management strategy, based on the current WHO guidelines, has been published elsewhere [27]. During screening by the nurses in the clinic and in the communities, women with a cervical leukoplakia finding on VIA/mobile colposcopy (Fig 2) were initially referred for gynaecologist evaluation at the CCPTC and for possible biopsy and histopathology evaluation (Fig 3). Women with detected cervical lesions were offered the option of biopsies, conservative management with follow-ups (if the lesions were minor changes/low-grade and the women were not likely to be lost to follow-up) and treatment with thermal ablation or loop electrosurgical excision procedure (LEEP). Clinically significant lesions were treated on-site using a handheld thermal coagulator (MTA-100 Thermal Ablation System; Liger Medical, USA), according to strict guidelines [30]. Eligibility for thermal coagulation was assessed based on VIA/EVA mobile colposcopy findings, with the following pre-specified outcomes: no suspicion of invasive disease; or entirely visible lesions without endocervical extension; or TZ type 1 lesions; or TZ type 2 lesions (if the tip of the thermal coagulator probe could reach the upper limit of the lesion). Clients with mild acetowhitening (minor changes) on colposcopy who were not likely to be lost to follow-up were counselled on the option of conservative management (rescreening in 6 months to 1 year) as these lesions would usually regress spontaneously. Women who tested negative for hr-HPV and showed no cervical abnormalities on visual inspection were advised to rescreen after 5 years (3 years if they were HIV positive). Women who tested hr-HPV positive without accompanying cervical lesions were counselled to repeat HPV DNA testing in 1 year. Notably, women in these last two categories received counselling after their HPV DNA tests were returned, not on-site, ensuring a more personalized and informed approach to their care.

### Statistical analysis

We report counts and percentages for all categorical sociodemographic and clinical variables of the women. Continuous variables are reported as means with standard deviations (SDs) for symmetric data and medians with interquartile ranges (IQRs) for skewed data. The overall prevalence rates of leukoplakia are reported as percentages with their 95% confidence intervals (CIs). Missing data were excluded listwise from regression models. Sensitivity analyses were not performed. A Chi-squared test was used to assess the association between absence or presence of leukoplakia and selected screening outcome variables. We further explored the risk factors for leukoplakia using univariate and multivariable logistic regression stratified by method of visual screening. The multivariable analysis was performed using the forward elimination procedure with an arbitrary threshold of p-value = 0.25 to select variables. We assessed multicollinearity using the variance inflation factor (VIF). All VIF values were below 5, indicating no evidence of problematic multicollinearity. Effect sizes from these regression analyses are reported as odds ratios (ORs) and adjusted ORs (aORs) with their 95% CIs. All statistical analyses were performed using Stata version 18.0 (StataCorp LLC, College Station, TX, USA). All hypothesis tests were two-tailed and conducted at a 5% significance level.

### Ethical considerations

This study utilised secondary data from the Catholic Hospital, Battor, which consisted entirely of fully anonymised records on leukoplakia cases. Given that this research involved only de-identified secondary data with no interaction with human participants and no foreseeable risks to privacy, anonymity, or individual reputations, it was determined to be exempt from rigorous ethical review in accordance with the principles outlined in the Declaration of Helsinki and later amendments. However, the CCPTC obtained written approval from the Hospital’s Ethics Review Board (CHB-ERC 002/01/24) to examine anonymized clinical records for research purposes. No informed consent was required, as the data were pre-existing, anonymized, and not collected specifically for this study. All analyses adhered to the terms and conditions of the data source to ensure ethical use and data integrity.

## Results

### Sociodemographic and clinical characteristics

The mean age of the women was 39.4 years (SD = 11.1), and the median parity was 1 (IQR: 0–3). Also, 51 women (0.40% 13,861 total screened) of the clients screened were less than 21 years old, with the youngest being 18 years old. The majority of participants identified as Christian (n = 11,235; 87.5%). Most women were married or had a steady partner (n = 6,298; 55.1%). Regarding educational attainment, 5,317 (51.4%) had secondary education or better. One in five women did not earn a wage and only 2,329 (22.5%) of participants indicated a monthly income of> GHC 500 (approximately $40 as of August 2025), with most women (n = 9,028; 70.3%) enrolled on the national health insurance scheme (NHIS) (S1 Table).

An HIV positive status was documented in 979 (7.6%) of participants. Hypertension was the most common chronic condition reported (n = 1,380; 10.8%), followed by sickle cell disease (n = 260; 2.0%) and asthma (n = 258; 2.0%). Notably, 1,373 (10.7%) participants reported a history of cervical screening and treatment (with, LEEP or thermal/cryo-ablation) (n = 30; 0.2%) (S2 Table).

### Prevalence of leukoplakia and secondary screening outcomes

A total of 13,861 were available during the study window; 12,847 women underwent cervical screening using a visual inspection method, whereas a smaller subset (n = 1014; 7.3%) was screened using non-visual methods such as HPV DNA testing and cytology and did not include an assessment of leukoplakia (Fig 1). The greater majority underwent leukoplakia assessment as part of visual inspection methods in combination with high-risk HPV testing or cytological evaluation. We found an overall leukoplakia prevalence of 0.9% (n = 120; 95% CI: 0.7–1.1). Out of 10,222 women screened by VIA, 0.2% (n = 23; 95% CI: 0.1–0.3) had a finding of leukoplakia compared to 3.7% (n = 97; 95% CI: 3.0–4.5) among women screened by Enhanced Visual Assessment (EVA) (Fig 1).

Detailed primary and secondary screening outcomes of interest for women who underwent cervical screening are presented according to visual inspection method in Table 1. The prevalence of leukoplakia among HIV+ women screened using VIA was 0.7% (95% CI: 0.2–2.1) whiles women screened using EVA was 6.1% (95% CI: 4.3–8.4). The prevalence of leukoplakia among hrHPV+ women screened using VIA was 1.1% (95% CI: 0.7–1.9), compared to 5.4% (95% CI: 3.8–7.4) of hrHPV+ women screened using EVA (Table 1). The majority (85.9%) of women screened using VIA exhibited type 3 transformational zone (TZ3). Similarly, 64.3% of women screened using EVA had a TZ3. Regarding HPV test results, 1,314 (18.5%) women screened with VIA tested positive for high-risk HPV compared to 616 (32.5%) who tested positive for high-risk HPV among those screened with EVA (Table 1).

**Table 1:** Screening outcomes for women according to visual inspection method (n = 12,847).

Variables	Estimate		
**Primary outcome**	**VIA**	**EVA**	**Total**
Leukoplakia, % (95% CI)	0.2 (0.2–0.3)	3.7 (3.0–4.5)	0.9 (0.8–1.1)
Leukoplakia in HIV+ women, % (95% CI)	0.7 (0.2–2.1)	6.1 (4.3–8.4)	3.6 (2.6–4.9)
Leukoplakia in high-risk HPV+ women % (95% CI)	1.1 (0.7–1.9)	5.4 (3.8–7.4)	2.5 (1.9–3.3)
**Secondary outcome**			
**TZ type on visual inspection, n (%)**			
TZ 1	402 (3.9)	324 (12.3)	726 (5.6)
TZ 2	975 (9.5)	581 (22.1)	1,556 (12.1)
TZ 3	8,782 (85.9)	1,688 (64.3)	10,470 (81.5)
Missing	61 (0.6)	34 (1.3)	95 (0.7)
**HPV testing done, n (%)**			
Yes	7,116 (69.6)	1,894 (72.1)	9,010 (70.1)
No	2,980 (29.2)	706 (26.9)	3,684 (28.7)
Missing	124 (1.2)	27 (1.0)	151 (1.2)
**HPV test results, n (%), n = 9010**			
High risk HPV negative	5,761 (80.9)	1,264 (66.7)	7,025 (78.0)
High risk HPV positive	1,312 (18.4)	615 (32.5)	1,927 (21.4)
Invalid	42 (0.6)	15 (0.8)	57 (0.6)
**Cytology test results, n = 636**			
Negative	75 (97.4)	537 (96.1)	612 (96.2)
ASC-US	1 (1.3)	7 (1.3)	8 (1.3)
Low grade (LSIL)	0 (0.0)	2 (0.4)	2 (0.3)
High grade (HSIL)	0 (0.0)	1 (0.2)	1 (0.2)
Other	0 (0.0)	1 (0.2)	1 (0.2)
Unsatisfactory	1 (1.3)	11 (2.0)	12 (1.9)

#TZ1: The entire circumference of the squamocolumnar junction is visible; completely ectocervical. TZ2: The entire circumference of the squamocolumnar junction is visible; partly or fully endocervical. TZ3: The entire circumference of the squamocolumnar junction is not visible; partly or fully endocervical.

Leukoplakia occurred more frequently in hr-HPV positive women (2.5%; 95% CI: 1.9–3.3) than hr-HPV negative women (0.7%; 95% CI: 0.5–0.9), and in HIV positive (3.6%; 95% CI: 2.6–4.9) versus HIV negative (0.8%; 95% CI: 0.6–1.1) (Table 2). leukoplakia was rare among women with abnormal cytology (0.0% (95% CI: 0.0–0.1) in low grade squamous intraepithelial lesions (LSIL) [0/2] and ASC-US [0/8] subgroups). (Table 2).

**Table 2:** Leukoplakia and screening outcomes for women (n = 12,847).

	Leukoplakia Prevalence % (95% CI)	P-value
**HPV test results**		
hr-HPV positive	2.5 (1.9–3.3)	<0.001
hr-HPV negative	0.7 (0.5–0.9)	
**Cytology test results**		
Negative	0.2 (0.1–0.3)	0.003
ASC-US	0.01 (0.0–0.1)	
Low grade (LSIL)	0.01 (0.0–0.1)	
High grade (HSIL)	0.0 (0.0–0.0)	
**HIV status**		
HIV negative	0.8 (0.6–1.1)	<0.001
HIV positive	3.6 (2.6–4.9)	

### HPV genotypes by method of testing

A total of 98 clients with leukoplakia were screened for high-risk HPV using four diagnostic platforms. Of these, 47 (48.0%) were tested with AmpFire, 34 (34.7%) with the MA-6000, 6 (6.1%) with GeneXpert, and 11 (11.2%) with the CareHPV platform. The proportion testing positive for high-risk HPV was 40.4% with AmpFire, 55.9% with MA-6000, 16.7% with GeneXpert, and 81.8% with CareHPV. Overall, HPV18 was the most frequently detected genotype, with a prevalence of 29.2% (95%CI: 17.9–43.8). Of the HPV18 cases, 8 (42.1%) were identified using the AmpFire platform and 6 (31.6%) using the MA-6000 system (Table 3).

**Table 3:** High Risk HPV Positive Genotypes by Method of testing.

HPV genotypes	Testing Platforms				Total = 98 n, % (95% CI) hr-HPV+ = 48, 49.0 (39.1–58.9) hr-HPV- = 49, 50.0 (40.1–59.9)
	*Ampfire = 47 n (%) hr-HPV+ = 19 (40.4) hr-HPV- = 27 (57.5)	MA-6000 = 34 n (%) hr-HPV+ = 19 (55.9) hr-HPV- = 15 (44.1)	Gene Xpert = 6 n (%) hr-HPV+ = 1 (16.7) hr-HPV- = 5 (83.3)	CareHPV = 11 hr-HPV+ = 9 (81.8) hr-HPV- = 2 (18.2)	
HPV 16	2 (10.5)	2 (10.5)	1 (100.0)	N/A	5, 10.4 (6.8 - 27.5)
HPV 18	8 (42.1)	6 (31.6)	–	N/A	14, 29.2 (17.9 - 43.8)
HPV 31	–	1 (5.3)	–	N/A	1, 2.1 (0.3 - 14.0)
HPV 33	–	1 (5.3)	–	N/A	1, 2.1 (0.3 - 14.0)
HPV 35	–	1 (5.3)	–	N/A	1, 2.1 (0.3 - 14.0)
HPV 45	–	1 (5.3)	–	N/A	1, 2.1 (0.3 - 14.0)
HPV 51	–	1 (5.3)	–	N/A	1, 2.1 (0.3 - 14.0)
HPV 56	–	2 (10.5)	–	N/A	2, 4.2 (1.0 - 15.7)
HPV 58	–	1 (5.3)	–	N/A	1, 2.1 (0.3 - 14.0)
HPV 31, 33, 35, 39, 45, 51,52, 53, 56,58, 59, 66, and 68 (combined).	13 (68.4)	12 (63.2)	–	N/A	25, 52.1 (37.8–66.0)
HPV 16, 18, 31, 33, 35, 39, 45, 51,52, 56, 58, 59, 66, and 68 (combined)	N/A	N/A	N/A	9 (81.8)	81.8 (44.1–96.3)

*One result is invalid.

### HPV genotypes by leukoplakia with or without acetowhitening

Among leukoplakia clients who tested positive for high-risk HPV, 45 (93.8%) did not exhibit acetowhitening, whereas 3 (6.3%) showed acetowhitening. Among those without acetowhitening, 13 (28.9%) were positive for HPV18, 4 (8.9%) for HPV16, and 23 (51.1%) for the combined group of other high-risk HPV types (31, 33, 35, 39, 45, 51, 52, 53, 56, 58, 59, 66, and 68) (Table 4).

**Table 4:** HPV Genotypes by Leukoplakia with or without acetowhitening.

HPV types	Leukoplakia	
	Leukoplakia without acetowhitening, n = 45	Leukoplakia with acetowhitening, n = 3
HPV 16	4 (8.9)	1 (33.3)
HPV 18	13 (28.9)	1 (33.3)
HPV 31	1 (2.2)	–
HPV 33	1 (2.2)	–
HPV 35	1 (2.2)	–
HPV 45	1 (2.2)	–
HPV 51	1 (2.2)	–
HPV 56	1 (2.2)	1 (33.3)
HPV 58	1 (2.2)	–
HPV 31, 33, 35, 39, 45, 51,52, 53, 56,58, 59, 66, and 68 (combined).	23 (51.1)	2 (66.7)
HPV 16, 18, 31, 33, 35, 39, 45, 51,52, 56, 58, 59, 66, and 68 (combined)	9 (20.0)	0 (0.0)

### Factors associated with leukoplakia

In the univariate logistic regression analysis of women screened with VIA, women who had never smoked (OR:0.02; 95% CI: 0.00–0.09; p-value < 0.001) were significantly associated with reduced odds of leukoplakia. However, women with high-risk HPV positive results were associated with increased odds of leukoplakia (OR: 13.25; 95% CI: 4.81–36.53; p-value <0.001). None of the other variables of interest were statistically significantly associated with leukoplakia in women screened with VIA. In the multivariate analysis, positive HIV status (aOR: 8.27; 95% CI: 1.18–57.69; p-value = 0.033), high-risk HPV positivity (aOR: 30.27; 95% CI: 3.56–257.37; p-value = 0.002), were associated with an increased odds of leukoplakia among women screened with VIA (S2 Table).

In the univariate logistic regression analysis of women screened with EVA, positive HIV status (OR=1.98; 95% CI:1.19–3.32; p-value = 0.009) and out-of-pocket payments (OR=1.61; 95% CI:1.03–2.52; p-value = 0.039) were significantly associated with increased odds of leukoplakia. In the multivariate analysis however, having a steady partner (OR: 3.38; 95% CI: 1.22–9.38; p-value = 0.019), and health insurance coverage (OR: 4.82; 95% CI: 1.09–21.20; p-value = 0.038) were associated with increased odds of leukoplakia whereas advanced level of education was found to be associated with decreased odds of leukoplakia (aOR: 0.24; 95% CI: 0.07–0.79; p-value = 0.019) (S2 Table).

### Referral outcomes for women with leukoplakia

**Fig 4:**
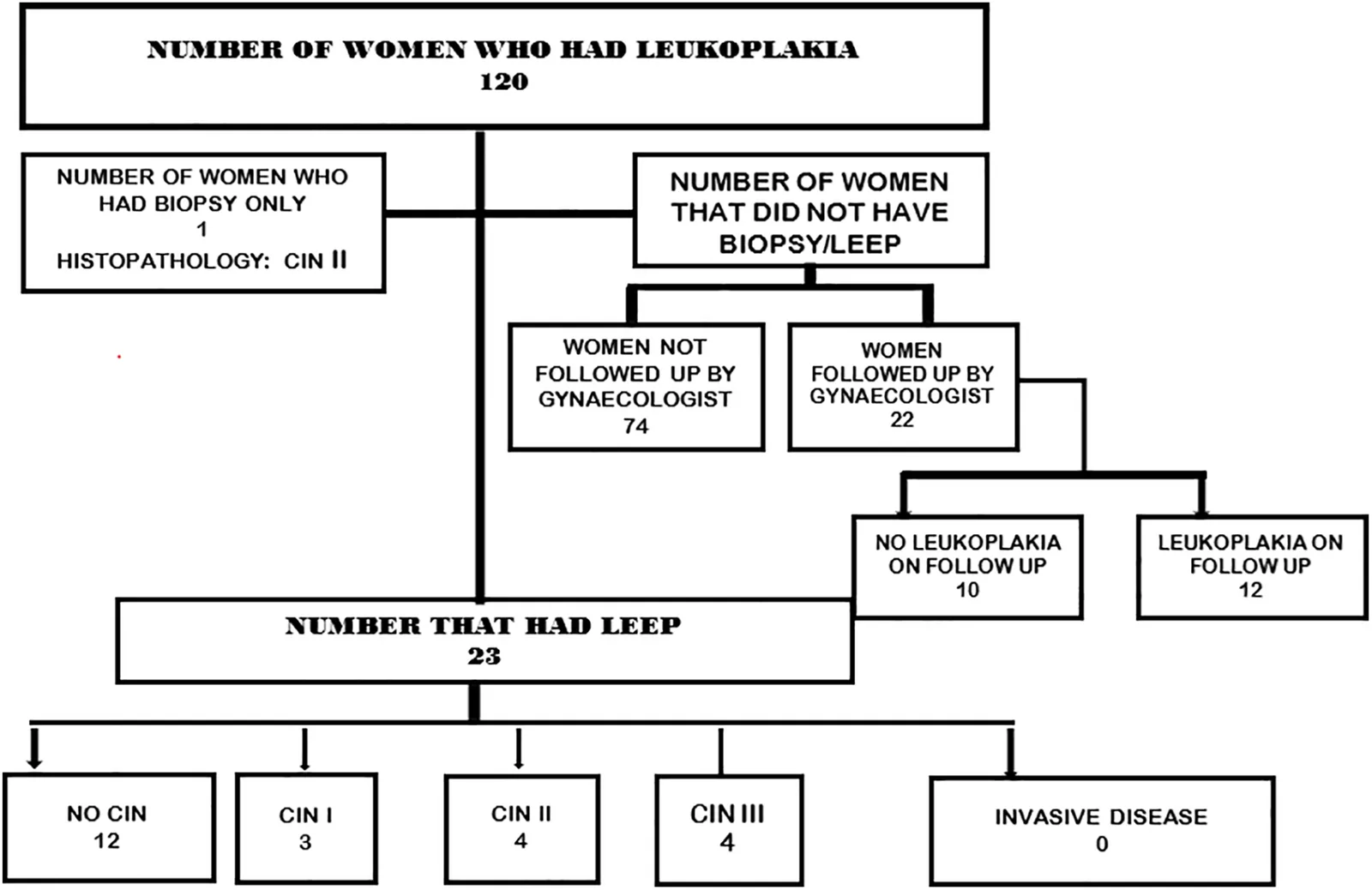
Histopathology outcomes among women with cervical leukoplakia, stratified by presence or absence of acetowhitening on visual inspection (VIA/EVA mobile colposcopy). Of 120 women with leukoplakia, 112 (93.3%) had no acetowhitening and 8 had acetowhitening on VIA/EVA. Histopathology results (n = 24 women who underwent biopsy/LEEP) are shown for the overall group and stratified subgroups. No invasive cancer was detected in any case.

Out of 120 women who were referred to the gynaecologist for management of leukoplakia, 74 (61.7%) were lost to care, yielding an initial follow-up success rate of 38.3% (n = 46/120) (Fig 3). Among 46 women who reported for a gynaecologist visit, a finding of leukoplakia was ruled out for 10 (21.7%) and the remaining 36 had biopsy/excision of the leukoplakia for histopathology recommended for them. A further 12 (33.3%) women were lost to care and did not have the biopsy/excision for histopathology. Of the 24 women with histopathology data, 12 women recorded no Cervical Intraepithelial Neoplasia (CIN) and were counselled appropriately, whereas Cervical Intraepithelial Neoplasia was reported as follows: CIN I: 3; CIN II: 5; CIN III: 4 (Fig 4).

**Fig 5:**
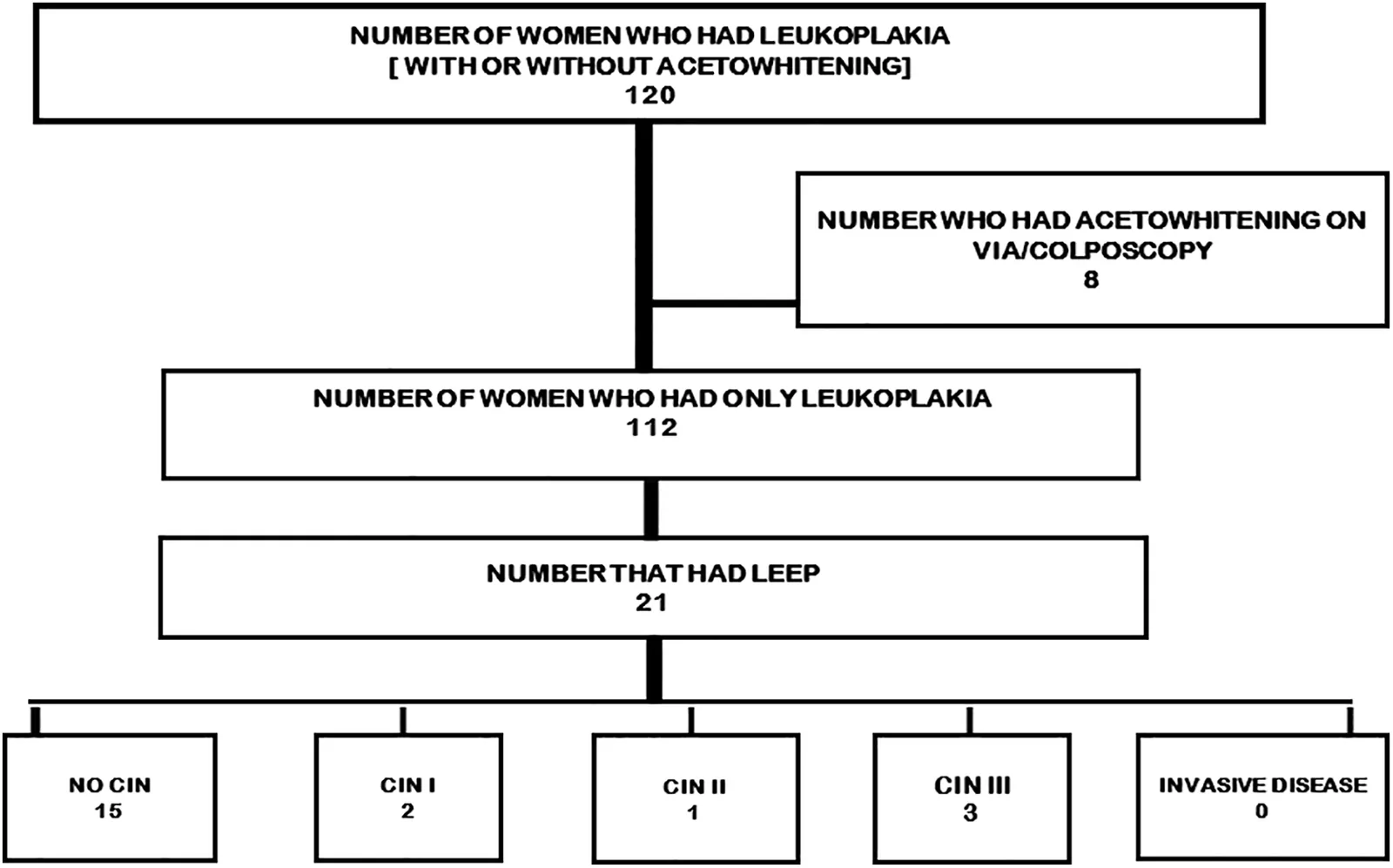
Histopathology outcomes among the 112 women with cervical leukoplakia who showed no acetowhitening on VIA or mobile colposcopy. Twenty-one women (18.8%) underwent LEEP. Histopathology results showed no dysplasia in 15 cases, CIN I in 2, CIN II in 1, and CIN III in 3. No invasive disease was identified. The remaining women did not undergo biopsy/LEEP, primarily due to loss to follow-up or financial barriers.

Of the 120 women with leukoplakia, we tracked histopathology outcomes based on the presence or absence of acetowhitening (Figs 4 and 5). For 112 (93.3%) who did not have acetowhitening on VIA/mobile colposcopy (Fig 5), 21 (17.5%) had LEEP. The histopathology results were: no CIN (15), CIN I (2), CIN II (1), and CIN III (3). None had invasive disease.

## Discussion

Real-world reporting on leukoplakia in cervical cancer screening is limited, as it is not a primary focus of cervical cancer screening studies or programmes in low-resource settings. This is the first study to examine the distribution of cervical leukoplakia disaggregated by visual inspection methods and associated factors as well as referral outcomes in a primary care setting. In addition, by assessing the clinical implications of cervical leukoplakia reporting in low-resource settings, our findings aim to guide evidence-based improvements in cervical cancer screening protocols for fragile health systems.

The marked difference in leukoplakia prevalence between screening methods is concordant with studies demonstrating disparate visual screening outcomes according to screening technology [13,31–33]. A higher leukoplakia prevalence observed with EVA (3.7%) compared to VIA (0.2%) supports evidence for EVA’s enhanced visualization and magnification capabilities, which improve detection of subtle epithelial changes [33]. Although primary HPV screening is the recommended approach, visual inspection methods have become the mainstay of cervical screening programmes wherever resources are stretched and lower implementation costs are desired. Our data suggests that visual assessment with some magnification offers superior sensitivity for identifying leukoplakia, which may be missed, particularly, in high-risk groups such as hrHPV-positive or HIV-positive women and calls for widespread refresher training in mobile colposcopy for healthcare staff involved in cervical screening based on visual methods.

The histopathological importance of leukoplakia is an important question in screening practice. Is isolated leukoplakia important clinically, or must it be associated with acetowhitening to be clinically important? Although the diagnostic importance of leukoplakia is uncertain, our data suggest that leukoplakia does not need to coexist with acetowhitening to be clinically important and confirms previous evidence of a strong association with Cervical Intraepithelial Neoplasia and high-risk HPV infections, signaling latent pathology [10,33]. This presents difficulties in low-resource environments, where excisional biopsies and histopathology services are frequently unattainable, and visual inspection is the predominant diagnostic approach [34]. In practice, this situation raises a need for practical field-adapted novel technologies and algorithms backed by evidence from prospective studies that can guide health workers in managing leukoplakia when histological confirmation is not immediately available.

EVA’s higher detection rate for leukoplakia risks overdiagnosis, as approximately 22% of suspected cases were ruled out as leukoplakia on specialist gynaecologist review (no lesion or benign findings). This raises concerns about misclassification and unnecessary treatment, particularly when not all leukoplakia represents serious pathology. In contrast, VIA’s lower sensitivity may miss high-grade lesions, delaying critical diagnoses. These experiences highlight the need for improved diagnostic tools in low-resource settings [13,33]. However, high rates of loss to follow-up and limited histologic confirmation impair our ability to conclusively answer this question based on our data. In limited settings, where patients often face barriers to ongoing care, screen-and-treat approaches can balance these risks by prioritizing immediate action, guiding clinical practice despite diagnostic uncertainties. In the near future, agentic artificial intelligence systems could be employed to enhance the predictive power of visual inspection imagery and streamline patient management.

Although the diagnostic value of leukoplakia is uncertain, its occurrence as a screening outcome alters the trajectory of patient management and poses significant challenges in low-resource settings in favour of diagnostic accuracy [13,35]. Leukoplakia typically requires further evaluation (biopsy/excision) before considering treatment due to the risk of underlying high-grade lesions [27,36–39]. Moreover, histopathology services are often unavailable in resource-constrained environments, delaying diagnosis, obfuscating treatment and increasing the risk of disease progression. Furthermore, screening in these settings is typically performed by low- and middle-cadre staff untrained in biopsy or excision procedures. Even when biopsies are feasible, the absence of haemostatic agents such as Monsel’s solution or silver nitrate in basic and resource-limited settings complicates the management of post-biopsy bleeding, as these resources are either unavailable or prohibitively expensive in fragile health systems [12,36]. Our experience is that leukoplakia disrupts screen-and-treat strategies, which rely on single-visit diagnosis and treatment to maximise compliance [12]. Women with leukoplakia require multiple visits for biopsy, histopathology, and follow-up, increasing the likelihood of loss to follow-up, particularly in rural areas where access to higher-cadre staff is limited. This challenge is compounded by the logistical barriers faced by women requiring screening services in low-resource settings, such as travel costs and livelihood constraints [40]. Agentic artificial intelligence systems could further strengthen visual inspection by standardising leukoplakia detection, reducing operator-dependent variability, and providing real-time risk stratification to guide triage decisions in settings where specialist review is limited.

Concurrent testing approaches, combining VIA or EVA with point-of-care molecular diagnostics (hrHPV testing, E6/E7 Oncoprotein tests/methylation tests), may improve risk stratification by identifying women with leukoplakia who are hrHPV-positive and thus require urgent referral. Such strategies could reduce unnecessary procedures while prioritizing high-risk cases, aligning with the WHO recommendations for integrated screening protocols [41]

Further, the high prevalence of TZ3 types represents another challenge in detecting abnormalities in this cohort using visual inspection methods. TZ3, defined by the partial or complete invisibility of the squamocolumnar junction (SCJ) in the endocervical canal, frequently conceals lesions and diminishes the diagnostic precision of VIA and colposcopy [42]. This discovery points to the necessity for supplementary interventions, including pharmaceutical treatments like misoprostol to enhance visualization: where a single dose of 400 mcg vaginal misoprostol has been shown to improve TZ visibility, as suggested in recent literature [43]. Improving the visibility of the TZ in these instances may elevate detection rates and diminish the likelihood of overlooking high-grade lesions.

We found that high-risk HPV positivity was a significant risk factor for leukoplakia, among VIA-screened women. This association points to the potential role of HPV-driven epithelial changes in the aetiogenesis of leukoplakia, consistent with its established oncogenic potential at other anatomical sites [44–46]. However, it’s worth noting that HPV DNA testing was not conducted on all women with leukoplakia; some underwent screening solely through a visual inspection approach. Furthermore, as partial genotyping was predominantly used for HPV DNA testing, pinpointing the specific HPV genotypes linked to leukoplakia could be challenging. Nevertheless, the association between hrHPV and leukoplakia presents a case for concurrent screening approaches which combine VIA/mobile colposcopy and hrHPV screening in a single visit [17,47]. In the present study, HIV-positive status was associated with increased odds of leukoplakia, likely due to immune suppression facilitating persistent HPV infection [48]. The association with marital status indicates that leukoplakia may be driven primarily by a complex interaction between both biological and demographic factors [45]. In the EVA group, women who reported having a steady partner showed significantly higher odds of leukoplakia. This demographic association likely reflects increased cumulative exposure to HPV through sexual activity within stable partnerships, and the complex interplay between persistent infection and relationship patterns.

The public health implications of these findings are profound. The disruption to the continuum of care caused by leukoplakia and its association with hrHPV and HIV reveals the need for a shift towards primary HPV screening with mobile colposcopy-based triaging for same-visit intervention. Training programs to equip low- and middle-cadre staff with basic biopsy skills are warranted. Again, proficiency in innovative fabric-tipped devices for endocervical curettage and similar devices that provoke minimal bleeding would be useful in screening programmes in low-resource settings. The provision of affordable hemostatic agents could enhance diagnostic capacity even further. Future research should focus on elucidating the histopathological correlates of leukoplakia to clarify its prognostic value, potentially through prospective studies that integrate molecular diagnostics. Additionally, evaluating cost-effective tools, such as AI-enhanced mobile colposcopy, could enhance leukoplakia detection and management, thereby improving the diagnostic sensitivity of direct visualisation methods. Prospective studies on leukoplakia are important to determine the cases that regress spontaneously. These studies may shed light on which cases of leukoplakia require urgent attention and which of them may regress. Interventions to reduce loss to follow-up, such as community-based follow-up systems, are also critical to ensure equitable access to cervical cancer prevention.

### Limitations

Given that leukoplakia was identified by multiple cadres and 21.7% of suspected cases were not seen at specialist review, the present leukoplakia label may reflect measurement variability across operators and modalities. At the CCPTC, for all positive cases seen at VIA, mobile colposcopy is used to capture images for quality assurance to ensure that cases of leukoplakia are well documented based on a standardized case definition. This may not be so in other settings without quality assurance protocols, making it difficult to tell if cases of leukoplakia seen by middle cadre staff on the field but absent on review by a gynaecologist represent true cases of leukoplakia. Because high-risk HPV positivity and genotype coverage differ across platforms and specimen types, aggregated genotype prevalence and hrHPV–leukoplakia associations may be influenced by assay heterogeneity. We present platform-stratified results to address this risk. In platform-stratified analyses, genotype distributions varied; aggregated HPV 16 and 18 prevalence estimates are platform-dependent and should be interpreted with caution. Finally, we did not perform an a priori sample size estimation because the data was primarily collected as part of routine service delivery; thus, the sample size was determined by the operational reach of the initiative instead of research-driven statistical requirements.

## Conclusion

This study directly demonstrates that cervical leukoplakia is a clinically important finding independent of acetowhitening, with a prevalence of 0.9% that varies markedly by screening method (0.2% with VIA vs. 3.7% with EVA mobile colposcopy) and is strongly associated with high-risk HPV positivity and HIV infection. It further documents real-world referral outcomes, revealing a 61.7% loss-to-follow-up rate and histopathological confirmation of Cervical Intraepithelial Neoplasia in a substantial proportion of biopsied cases. These results amplify critical gaps between enhanced detection capacity and effective care delivery in resource-constrained settings and assert the need for simplified, decentralised management protocols that integrate point-of-care HPV testing with mobile colposcopy triage

To accelerate progress toward cervical cancer elimination in low- and middle-income countries, stakeholders must prioritise the development and implementation of clear community-level guidelines for leukoplakia management, accessible molecular diagnostics, and AI-supported visual inspection tools to reduce operator variability and enhance leukoplakia detection. Only through such targeted innovations can the diagnostic and logistical challenges identified in this Ghanaian training centre be transformed into equitable, sustainable screening strategies.

## Supporting information

S1 Table Sociodemographic and clinical characteristics of women who underwent cervical screening via visual inspection method (n = 12,847). (DOCX)

S2 Table Exploratory logistic regression analysis of sociodemographic and clinical factors associated with leukoplakia among women who underwent cervical screening via visual inspection method using acetic acid and mobile colposcopy. (DOCX)

S1 File Raw data file. (RAR)

## Abbreviations

aOR: adjusted odds ratio
CCPTC: Cervical Cancer Prevention and Training Centre
CI: confidence interval
EVA: Enhanced Visual Assessment
FHW: female health worker
hr-HPV: high-risk human papillomavirus
IFCPC: International Federation of Cervical Pathology and Colposcopy
IQR: interquartile range
LEEP: loop electrosurgical excision procedure
NHIS: National Health Insurance Scheme
OR: odds ratio
SD: standard deviation
TZ: transformation zone
VIA: visual inspection with acetic acid
WHO: World Health Organization
