# Supplementary material for: Cervical leukoplakia in Ghana: Unveiling diagnostic challenges and informing low-resource screening strategies
Source: PLoS One. 2026 Sep 9;21(9):e0345847. doi: 10.1371/journal.pone.0345847 (PMC13557359; doi:10.1371/journal.pone.0345847)
Supplement: Supplementary file 1 [file pone.0345847.s001.docx]

S1 Table: Supplementary Table 1**:** Sociodemographic and clinical characteristics of women who underwent cervical screening via visual inspection method (n=12,847)

| **Variables** | | **Estimate** |
| --- | --- | --- |
| **Sociodemographic variables** | |  |
| Age, years: mean (SD) | | 39.4 (11.0) |
| Parity, median (IQR) | | 1 (0 – 3) |
| Religion, n (%) | |  |
|  | Christianity | 11,235 (87.5) |
|  | Islamic | 609 (4.7) |
|  | Traditional | 105 (0.8) |
|  | Other | 48 (0.4) |
|  | Missing | 850 (6.6) |
| Marital Status, n (%) | |  |
|  | Single | 1,243 (9.7) |
|  | Has a steady partner | 1,870 (14.6) |
|  | Married | 4,428 (34.5) |
|  | Divorced | 587 (4.6) |
|  | Widowed | 599 (4.7) |
|  | Missing | 4,120 (32.1) |
| Education, n (%) | |  |
|  | No formal education | 1,070 (8.3) |
|  | Elementary education | 1,238 (9.6) |
|  | Secondary education | 3,607 (28.1) |
|  | Tertiary education | 1,710 (13.3) |
|  | Other | 317 (2.5) |
|  | Missing | 4,905 (38.2) |
| Earn an income, n (%) | |  |
|  | Yes | 10,353 (80.6) |
|  | No | 2,138 (16.6) |
|  | Missing | 366 (2.8) |
| Monthly Income^¥^, n (%) | | |
|  | <100 | 1,063 (10.3) |
|  | 100-250 | 1,438 (13.9) |
|  | 250-500 | 1,519 (14.7) |
|  | >500 | 2,329 (22.5) |
|  | Unable to say | 435 (4.2) |
|  | Missing | 3,569 (34.5) |
| Source of funds for medical bill payment^∞^ | |  |
|  | Self, n (%) |  |
|  | Yes | 7,042 (54.8) |
|  | No | 5,805 (45.2) |
| Relatives, n (%) | |  |
|  | Yes | 3,154 (24.5) |
|  | No | 9,693 (75.5) |
| Current or former employee, n (%) | |  |
|  | Yes | 212 (1.6) |
|  | No | 12,635 (98.4) |
| Health Insurance, n (%) | |  |
|  | Yes | 9,028 (70.3) |
|  | No | 3,819 (29.7) |
| Ever smoked, n (%) | |  |
|  | Yes | 22 (0.2) |
|  | No | 8,738 (68.9) |
|  | Missing | 4,087 (31.8) |
| Currently smokes, n (%) | |  |
|  | Yes | 22 (0.2) |
|  | No | 8,848 (68.9) |
|  | Missing | 3,977 (30.9) |
| Past contraceptive use, n (%) | |  |
|  | Yes | 2,626 (20.4) |
|  | No | 6,212 (48.4) |
|  | Missing | 4,009 (31.2) |
| Current contraceptive use, n (%) | |  |
|  | Yes | 760 (5.9) |
|  | No | 8,077 (62.9) |
|  | Missing | 4,010 (31.2) |
| **Clinical Characteristics** | |  |
| HIV status, n (%) | |  |
|  | Positive | 979 (7.6) |
|  | Negative | 4,690 (36.5) |
|  | Unknown | 7,019 (54.6) |
|  | Missing | 159 (1.2) |
| Chronic conditions | |  |
| Hypertension, n (%) | |  |
|  | Yes | 1,380 (10.8) |
|  | No | 11,467 (89.2) |
| Asthma, n (%) | |  |
|  | Yes | 258 (2.0) |
|  | No | 12,589 (98.0) |
| Sickle cell disease, n (%) | |  |
|  | Yes | 260 (2.0) |
|  | No | 12,587 (98.0) |
| Tuberculosis, n (%) | |  |
|  | Yes | 62 (0.5) |
|  | No | 12,785 (99.5) |
| Diabetes mellitus, n (%) | |  |
|  | Yes | 222 (1.7) |
|  | No | 12,625 (98.3) |
| Previous cervical screening, n (%) | |  |
|  | Yes | 1,373 (10.7) |
|  | No | 10,840 (84.4) |
|  | Missing | 634 (4.9) |
| Previous cervical treatment, n (%) | |  |
|  | Yes | 30 (0.2) |
|  | No | 8,762 (68.2) |
|  | Missing | 4,055 (31.6) |

*SD: Stand deviation; IQR: Interquartile range; HIV: Human immunodeficiency virus*

*^¥^Among 10,477 women who earn income*
