# Supplementary material for: Cervical leukoplakia in Ghana: Unveiling diagnostic challenges and informing low-resource screening strategies
Source: PLoS One. 2026 Sep 9;21(9):e0345847. doi: 10.1371/journal.pone.0345847 (PMC13557359; doi:10.1371/journal.pone.0345847)
Supplement: Supplementary file 2 [file pone.0345847.s002.docx]

S2 Table: Supplementary Table 2**:** Exploratory logistic regression analysis of sociodemographic and clinical factors associated with leukoplakia among women who underwent cervical screening via visual inspection method using acetic acid and mobile colposcopy

| **Variables** | | **VIA** | | **Mobile Colposcopy** | |
| --- | --- | --- | --- | --- | --- |
|  |  | OR [95% CI], p-value | AOR [95%CI], p-value | OR [95% CI], p-value | AOR [95%CI], p-value |
| Age Group | |  |  |  |  |
|  | <41 years | Ref | Ref | Ref | - |
|  | >=41 yrs | 0.38 [0.14 - 1.03], 0.057 | 0.11 [0.01 - 1.24], 0.075 | 1.11 [0.74 - 1.69], 0.608 | - |
| Parity | |  |  |  |  |
|  | <2 | Ref | - | Ref | Ref |
|  | 2 or more | 0.59 [0.25 - 1.38], 0.223 | - | 0.76 [0.50 - 1.14], 0.183 | 0.49 [0.22 - 1.12], 0.091 |
| Marital Status | |  |  |  |  |
|  | Single/Divorced/Widowed | Ref | - | Ref | Ref |
|  | Married/Has a steady partner | 1.88 [0.54 - 6.51], 0.318 | - | 1.29 [0.74 - 2.26], 0.369 | 3.38 [1.22 – 9.38], **0.019** |
| Education | |  |  |  |  |
|  | No formal education | Ref | - | Ref | Ref |
|  | Elementary education | 0.78 [0.21 - 2.95], 0.719 | - | 0.80 [0.39 - 1.66], 0.558 | 0.46 [0.14 - 1.49], 0.192 |
|  | Secondary/Tertiary education | 0.82 [0.21 - 3.30], 0.784 | - | 0.49 [0.24 - 1.02], 0.056 | 0.24 [0.07 - 0.79], **0.019** |
| Earn an income | | 0.87 [0.29 - 2.55], 0.794 | - | 1.50 [0.87 - 2.60], 0.142 | - |
| Source of funds for medical bill payment | |  |  |  |  |
|  | Self | 1.27 [0.55 - 2.93], 0.577 | 10.77 [0.92 – 126.60], 0.059 | 0.91 [0.61 - 1.37], 0.652 | - |
|  | Relatives | 1.03 [0.40 - 2.60], 0.957 | 3.33 [0.51 – 21.86], 0.210 | 1.61 [1.03 - 2.52], **0.039** | 1.82 [0.73 - 4.55], 0.199 |
|  | Insurance | 0.78 [0.33 - 1.85], 0.580 | - | 1.21 [0.76 - 1.91], 0.413 | 4.82 [1.09 - 21.20], **0.038** |
| Never smoked | | 0.02 [0.00 - 0.09], <**0.001** | - | - | - |
| Past contraceptive use | | 0.73 [0.24 - 2.22], 0.577 | - | 0.68[0.40 – 1.17], 0.167 | - |
| Current contraceptive use | | 0.65 [0.09 - 4.87], 0.674 | - | 0.23 [0.06 – 0.95], **0.042** | - |
| HIV status | |  |  |  |  |
|  | Negative | Ref | Ref | Ref | Ref |
|  | Positive | 3.60 [0.93 - 13.99], 0.064 | 8.27 [1.18 - 57.69], **0.033** | 1.98 [1.19 - 3.32], **0.009** | 1.99 [0.83 – 4.78], 0.126 |
| HPV result | |  |  |  |  |
|  | High risk HPV negative | Ref | Ref | Ref | - |
|  | High risk HPV positive | 13.25 [4.81 - 36.53], **<0.001** | 30.27 [3.56 - 257.37], **0.002** | 1.57 [0.99 - 2.49], 0.056 | - |
